# Ultrafast electrical switching of nanostructured metadevice with dual-frequency liquid crystal

**DOI:** 10.1038/s41598-019-55656-z

**Published:** 2019-12-30

**Authors:** Rafał Kowerdziej, Jerzy Wróbel, Przemysław Kula

**Affiliations:** 1 Institute of Applied Physics, Military University of Technology, 2 Kaliskiego Str., 00-908 Warsaw, Poland; 2 Institute of Physics, Polish Academy of Sciences, 32/46 Lotników Avenue, 02-668 Warsaw, Poland; 3 Institute of Chemistry, Military University of Technology, 2 Kaliskiego Str., 00-908 Warsaw, Poland

**Keywords:** Nanoscale materials, Soft materials, Metamaterials, Nanophotonics and plasmonics

## Abstract

Shortening of switching times of various soft-matter-based tunable metamaterials is one of the key challenges to improve the functionality of modern active devices. Here we show an effective strategy in the evolution of soft-matter-based tunable metamaterials that makes possible acceleration of both on and off switching processes by using a dual-frequency liquid crystal mixture. The frequency-convertible dielectric anisotropy of the dual-frequency mixture enabled us to create a fast-response in-plane switching metasurface at the nanoscale, which could be tuned by an electrical signal with different frequencies. The results clearly show that the resonance of the metamaterial can be continuously and reversibly controlled within a wavelength range of 100 nm as the applied frequency is inverted between 1 kHz and 40 kHz, with a total response time (τ = τ_ON_ + τ_OFF_) of 1.89 ms. Furthermore, experimental characteristics of the hybrid metamaterial are in great agreement with numerical calculations, which allow us to anticipate active epsilon-near-zero behavior of the metadevice. This work indicates the future development direction of liquid-crystal-based active plasmonic systems.

## Introduction

The “structuring” properties of liquid crystals (LCs) are very attractive for their assembly and control characteristics to design tunable plasmonic hybrids. Active LC-based metamaterials have been attracting special attention in the past few years because of their emerging applications in various photonic devices, for example, modulators, switches, and filters^1–6^. This attention is influenced by a number of advantages of LC-loaded plasmonic materials. Indeed, the use of LCs allows for the construction of metadevices with dynamic functions induced by electro-, magneto-, photo-, and temperature-sensitive properties^7–16^. These exotic properties have resulted in a significant increase in recent works devoted to application of LCs, and nematics especially, as an active component of hybrid metamaterials. For example, Buchnev *et al*. considered a near-infrared active metasurface functionalized with a nematic LC (NLC), which helped to overcome the problem of strong anchoring and involved, for the first time, design of electrically controlled nanoscale in-plane switching (IPS) of the LC molecular orientation^17^. Another important phenomenon resulting from structuring properties of liquid crystals, is stiction suppression in LC hybrid metamaterial nano-electro-mechanical systems, which was recently discovered by a group at Southampton^18^. Chikhi *et al*. proposed a hybrid reconfigurable metadevice based on LCs as a tuning element built from an array of split-ring resonators possessing conducting cantilevers in the gap region^19^. Lewandowski *et al*. described reversible tuning of structural and plasmonic properties of liquid-crystalline gold nanoparticle assemblies^20^. More recently, Wang *et al*. designed a tunable terahertz metamaterial absorber with graphene-assisted high-efficiency LCs^21^. Finally, our group has demonstrated several concepts of electrically and thermally tunable micro-structured metamaterials hybridized with highly birefringent NLCs^22–25^. These examples clearly show that nematics have been used for a long time in the implementation of switchable plasmonic systems. However, despite continuous progress, fast-response metadevices based on the NLC approach are extremely challenging to realize due to natural restrictions: their main disadvantage is a long switch-off time, that is, return (relaxation) of the director to the initial state after switching off the voltage. In fact, only the switch-on time can be shortened by applying a large electrical signal. The combination of these phenomena is a direct reason that switch-on time is much shorter than switch-off time (τ_ON_ ≪ τ_OFF_). Consequently, the response times are unsymmetrical and the total response time is long, which often degrades performance of LC-based electro-plasmonics. Thereby, an additional development towards fast-switching tunable LC-based plasmonic nanodevices is indispensable.

In this work, we report on overcoming these switching barriers by employing a dual-frequency method that equalizes the switching times (τ_ON_ and τ_OFF_) of electro-plasmonics as they are both voltage dependent, thereby also reducing the overall response time (τ = τ_ON_ + τ_OFF_)^26^. By embedding the nanoscale metamaterial into an in-plane-switching cavity filled with dual-frequency LC (DFLC), we have fabricated a dynamically switchable metadevice with submillisecond response to electrical stimuli, about three orders of magnitude lower compared to metasurfaces loaded with NLCs. Importantly, not only does the hybrid system presented here allow ultra-fast switching of plasmonic resonance, we found that it also exhibits tunable epsilon-near-zero (ENZ) properties. Recently, ENZ materials have become of particular interest because they allow for unprecedented control of phase manipulation of light, with a wide array of applications for novel light-matter interactions^27–29^. ENZ materials based on DFLC are especially interesting because they are characterized by ultrafast (<1 ms) response times, and they can be optimized relatively easily to work over the entire visible spectrum, which is crucial for novel optical technologies. In contrast to passive prototypes of such materials^30,31^, here we demonstrate a DFLC-based hybrid metamaterial that exhibits theoretically foreseen switchable ENZ properties and enables multifunctional uses. In addition, the designed DFLC mixture is characterized by a high birefringence that allows more efficient tuning of plasmonic properties^32,33^.

## Results and Discussion

**Figure 1:**
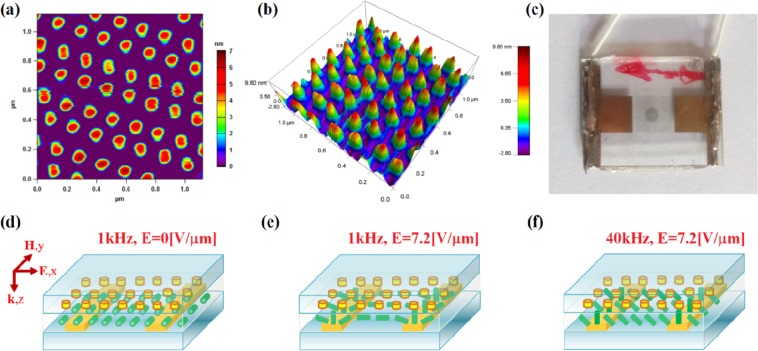
Fast-response electro-plasmonics: DFLC-metamaterial hybrid. (**a**) AFM image of nanostructured metasurface. (**b**) AFM 3D image of the topography of the metamaterial nanostructure. (**c**) Photo of the fabricated DFLC-metamaterial hybrid cell. Scheme of the IPS cell with nanostructured metamaterial when the molecules of the DFLC are driven by an electric field with (**d**) frequency of 1 kHz and amplitude E = 0 V/µm, (**e**) frequency of 1 kHz and amplitude E = 7.2 V/µm, and (**f**) frequency of 40 kHz and amplitude E = 7.2 V/µm.

The nanostructure design was based on periodically arranged metallic nano-islands that work as plasmonic resonators. Their spatial distribution and geometric dimensions obtained by atomic force microscopy (AFM) analysis are shown in Fig. 1(a,b). The nano-islands have a diameter of 100 nm and a height of only 6 nm to reduce ohmic losses in the hybrid metadevice. The whole structure is composed of unit cells having a square shape with sides measuring about 200 nm. A top-view image of the produced metadevice is presented in Fig. 1(c). To achieve fast and reproducible spectral tuning of plasmonic resonance, the metamaterial was hybridized with a 1.8-µm-thick layer of the DFLC mixture driven by the IPS mode, as shown in Fig. 1(d,f). In this type of cell, stripe electrodes are connected alternately to opposite ports to which the voltage is applied. The golden electrodes with a width of 10 μm and spacing of 20 μm have been etched using a commercial mix AU-5 from Cyantek. To ensure a planar orientation of LC molecules, internal surfaces of the IPS cell were coated with a thin film of uniformly rubbed polyimide Sunever SE-130 (Nissan Chemical Industries, Ltd.). Implementation of the described procedures allowed for the construction of the test cell shown in Fig. 1(c).

**Figure 2:**
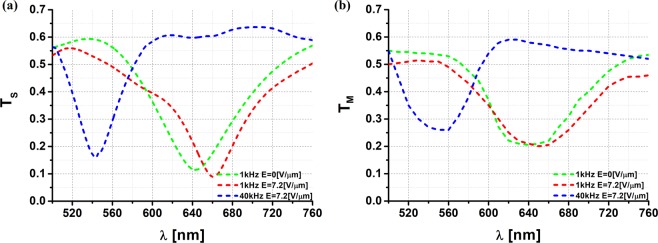
Active electrical tuning of nanoscale metasurface hybridized with dual-frequency liquid crystal mixture. (**a**) simulated (T_S_) and (**b**) measured (T_M_) transmittance spectra under applied electric field of 0 V/µm and 7.2 V/µm at 1 kHz, as well as 7.2 V/µm at 40 kHz.

To examine tunability and switching times of the fabricated DFLC-metamaterial hybrid, we first characterized transmittance spectra as a function of an external electric field, as shown in Fig. 2(a,b). For this purpose, a dual-frequency control signal – with the frequency switched between low frequency f_L_ = 1 kHz and high frequency f_H_ = 40 kHz was applied to the IPS electrodes of a hybrid LC cell to reorient the DFLC mixture. Figure 2(a) shows the simulated spectra of the tunable metadevice under the applied electric fields of 0 V/µm and 7.2 V/µm at 1 kHz, as well as 7.2 V/µm at 40 kHz. Please note that in our study, the electric field E = 1 V/µm corresponds to an applied voltage of U = 20 V. When no voltage is applied to the cell, the molecules of the DFLC mixture are aligned along the *y* direction, as shown in Fig. 1(d). In this case, the spectrum shows resonance at λ = 640 nm. When the driving signal at the frequency of 1 kHz gradually increases to E = 7.2 V/µm, the molecules are reoriented along the *x* direction, as shown in Fig. 1(e). As a result, the spectral peak is redshifted from 640 nm to 660 nm, as depicted in Fig. 2(a). Finally, when the frequency of the control signal is switched from 1 kHz to 40 kHz, and the value of the electric field does not change and is still E = 7.2 V/µm, the peak is blueshifted from 660 nm to 540 nm. The resonance tuning results from the fact that the DFLC mixture has positive dielectric anisotropy at low frequencies and negative dielectric anisotropy at high frequencies, that is, above the cross-over frequency, which in our case is approximately f_c_ = 12.6 kHz. The numerical analysis demonstrates that metamaterial resonance can be self-regulated by varying the applied voltage with respect to both the intensity (up to 7.5%) and spectrum (up to 120 nm), as evident from Fig. 2(a). Also, electromagnetic simulations have shown that intensity tunability can be improved up to 10.5% by increasing the thickness of the DFLC layer to 3 µm.

**Figure 3:**
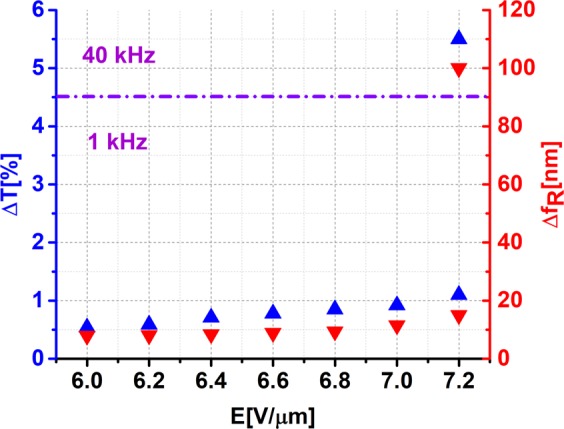
Transmittance intensity (ΔT) and resonance frequency shift (Δf_R_) of DFLC-loaded metasurface measured as functions of applied voltage.

The double electrical tuning mechanism was verified experimentally [see Fig. 2(b)]. The experimental results show qualitatively similar behavior; however, we measured a smaller resonance depth and smaller tuning of both the magnitude and resonant frequency. A shallower metamaterial resonance is primarily related to defects in the metasurface. Also, the simulation results give information about the absolute theoretical tunability limits. Indeed, in simulation processes, we consider only the metamaterial’s unit cell and use periodic boundary conditions on the planes that are orthogonal to *x* and *y*. Therefore, the metamaterial structure is expected to be periodic and spotless in the numerical simulation, which is not quite the case for the experimental sample, especially at the nanoscale. In the production of 10^8^ nanoelements, surface defects cannot be avoided. Also, the director’s profile in the IPS cell was not included in the numerical simulations. These explain the deviations in the resonance strengths and in the tunability between the numerical analysis and experimental results [see Fig. 2(a,b)]. In addition, theoretical transmittance curves have the same width, opposed to the experimental ones. This has to do with the fact that the calculated resonance width is constant, and its shift is related to the change of effective permittivity of the DFLC only. Practically, as the AC bias voltage rises, the permittivity of the surrounding medium (DFLC) is increased. This results in greater light scattering. Therefore, a red-shift and a widening of the resonance curve is observed^15,24^. Nevertheless, the experimental results clearly show that the metadevice can be tuned effectively, with an intensity change of up to 5.5% and spectrum up to 100 nm, just by changing the magnitude of the AC bias voltage, as evident from Fig. 3.

**Figure 4:**
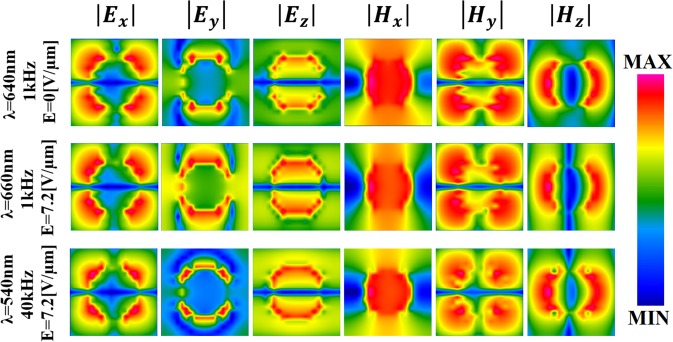
Spatial distribution of three components of the E-field and H-field for the three resonant frequencies at 640 nm, 660 nm, and 540 nm on the metasurface in the *x-y* plane. These frequencies are the result of a dual-frequency control pulse for which the applied frequency is switched between 1 kHz and 40 kHz. As a result, the DFLC molecules are reoriented.

These values are close to the absolute theoretical limit of 7.5% for intensity tunability and 120 nm for spectral tunability [see Fig. 2(a)]. In addition, we can observe that the strongest resonance occurs at the wavelength λ = 660 nm, a moderate resonance at λ = 640 nm, and the weakest at λ = 540 nm. To understand this phenomenon, the electric field and magnetic field spatial distributions at three resonance peaks, namely 640 nm, 660 nm, and 540 nm, were analyzed on the surface of the nano-island (see Fig. 4).

Mostly, these three resonances are stimulated by electric dipoles excited on the nano-islands for transverse-magnetic polarization. However, distributions of both electric and magnetic fields in the cross-section (*x-y* plane in Fig. 4) are undeniably different. The deepest resonance occurring at λ = 660 nm corresponds mainly to the electric field components |E_Y_| and |E_Z_|. In other words, the |E_Y_| and |E_Z_| components are in this case much stronger than at the other two wavelengths, that is, 640 nm and 540 nm, as shown in Fig. 4. Three components of the electric field and |E_X_| component of the electric field are responsible for the middle peak at λ = 640 nm. Otherwise, the weakest low-wavelength resonance at 540 nm is associated with the powerless components of the electric field, that is, |E_X_|, |E_Y_|, and |E_z_|, as well as the powerless component of magnetic field |H_Y_|. This weak impact is only to a small extent compensated by the component |H_Z_|, which is the strongest for this frequency. Therefore, we can attribute the feeble resonance at 540 nm to the combined effect of the electric field and magnetic field component along the *y* axis. Thus, the stronger the collective surface plasmon polariton resonance, the deeper the resonance.

**Figure 5:**
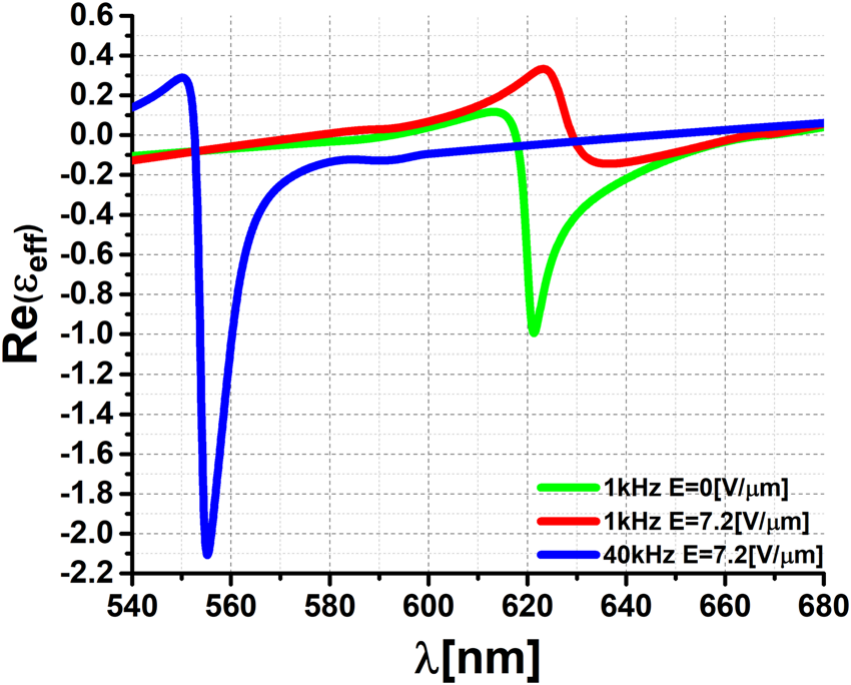
Theoretically predicted real part Re(ε_eff_) of the dielectric function of active metadevice under applied electric fields of 0 V/µm and 7.2 V/µm at 1 kHz, as well as 7.2 V/µm at 40 kHz.

The dielectric properties of the metadevice were also considered to achieve an ENZ regime (Fig. 5). The effective dielectric function (ɛ_eff_) was calculated for electric fields of 0 V/µm and 7.2 V/µm at 1 kHz, as well as 7.2 V/µm at 40 kHz, using the algorithm proposed by D. R. Smith^34^. As seen in the figure, the hybrid metamaterial exhibits a transition in the real part of the dielectric permittivity from positive to negative as a function of wavelength, such behavior of the real part of ɛ_eff_ is related to a Lorentzian resonance centered at the collective surface plasmon polariton resonance. This allows us to classify the designed hybrid system as ENZ. It is shown that by switching the frequency of a dual-frequency addressing signal and thus controlling the orientation of DFLC molecules, it is possible to induce a sharp and fast (on the same time scale as response times of the DFLC mixture) transition from metallic [Re(ɛ_eff_) < 0] to dielectric [Re(ɛ_eff_) > 0]. The ENZ state is only fulfilled for the real part of effective permittivity (as a result of absorption that will always entail that the imaginary part is higher than zero), and at one single wavelength (due to dispersion). Such unusual properties may either exist naturally at the plasma frequency or may result from arranging the propagation medium, for example so that light is spreading in a waveguide near the cutoff frequency^35^.

**Figure 6:**
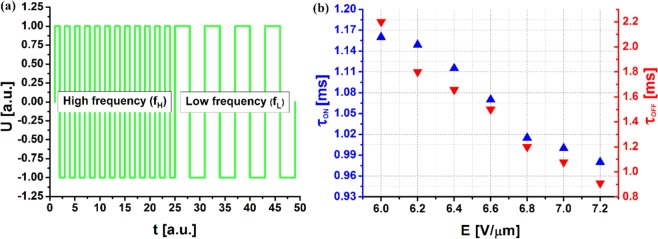
(**a**) Dual-frequency signal waveform U(t). (**b**) Response times τ_ON_ and τ_OFF_ of active metadevice in case of using a dual-frequency pulse switched between f_L_ = 1 kHz and f_H_ = 40 kHz.

In the next step, a dual-frequency control pulse was applied to the DFLC-loaded metadevice to test the switching times as a function of the applied voltage. The results of these experiments are presented in Fig. 6, where (a) a dual-frequency signal waveform U(t) and (b) response times τ_ON_ and τ_OFF_ are shown. The metadevice was placed in a birefractive system and illuminated with a halogen lamp employing a linear polarizer with neutral spectral characteristics. Importantly, low (f_L_ = 1 kHz) and high (f_H_ = 40 kHz) frequency signal sections had the same duration, t = 0.5 s. In Fig. 6(b), both the on and off times fall to values below 1 ms when the electric field increases to E = 7.2 V/µm. The exact value of the on and off switching times are τ_ON_ = 0.98 ms, and τ_OFF_ = 0.91 ms, respectively Therefore, according to the earlier results, the resonant frequency of the nanodevice can be continuously and reversibly tuned from λ = 640 nm to λ = 655 nm at a time of τ_ON_ = 0.98 ms, and from λ = 655 nm to λ = 555 nm at τ_OFF_ = 0.91 ms. Note that in the case of dual-frequency switching, response times are shortened because they are both voltage-dependent.

**Figure 7:**
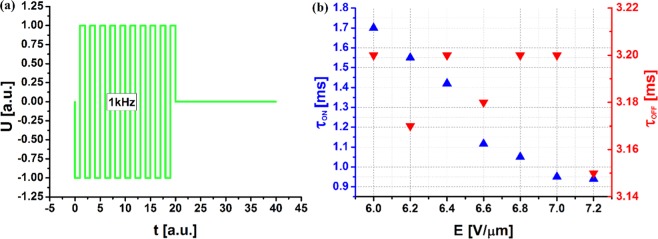
(**a**) Single-frequency signal waveform U(t). (**b**) Response times τ_ON_ and τ_OFF_ of active metadevice in case of using a 1-kHz single-frequency pulse.

To further emphasize the advantages of the dual-frequency switching and the nature of the plasmonic resonance tuning using a dual-frequency pulse, additional experiments were carried out employing a single-frequency pulse, with the frequency f = 1 kHz, as shown in Fig. 7(a,b). As expected in this case, only the switch-on time is shortened to the value of τ_ON_ = 0.94 ms as the applied voltage increases up to E = 7.2 V/µm. The off time is much longer, and fluctuates around τ_OFF_ ≈ 3.2 ms. Thus, only the switch-on time depends on the applied voltage, and the switch-off time depends on the relaxation time of the liquid crystal molecules.

## Conclusions

In summary, we have introduced the plasmonic architecture of a fast-response nanoscale metadevice loaded with a DFLC mixture, which enabled us to equalize the switching times (τ_ON_ and τ_OFF_) of electro-plasmonics because they are both voltage dependent. As a result, the achieved total response time (τ = τ_ON_ + τ_OFF_) was only 1.89 ms. Importantly, the resonant response of the constructed hybrid system could be tuned both in respect of its intensity (up to 5.5%) and wavelength (up to 100 nm) in a reversible and controllable way. The differences in the depth of resonances occurring for individual control pulses were clarified by means of electromagnetic simulations showing the spatial distributions of electric and magnetic fields around the nano-island. Also, we initiated a scheme of reversible switching of the ENZ regime by employing a dual-frequency addressing method in a plasmonic nanodevice. Therefore, the proposed design helped us to fully use the structural properties of soft matter as the fundamental element of a dynamic plasmonic system. It is worth pointing out that by limiting the height of nanoelements to about 6 nm and liquid crystal layer’s thickness to just 1.8 µm, we minimized the nanodevice’s ohmic losses. Relatively small losses, high tunability of both amplitude and resonant frequency, much faster switching times, and continuously switchable ENZ properties make our solution stand out from the previously published ones employing DFLC^36^. We believe that our fast-response DFLC-based active metamaterials are paving the way to numerous applications, including non-reciprocal magneto-optical effects, dielectric permittivity sensing, nonlinear ultrafast optical tuning, and self-assembled plasmonic systems^37–46^.

## Methods

### Numerical modeling

The full-wave numerical simulations were carried out using a general-purpose electromagnetic simulator QuickWave 3D, which has an algorithm based on the conformal finite-difference time-domain method. In the simulations, it was necessary to model only a unit cell of the metasurface with dimensions of 200 nm × 200 nm in the *x-y* plane. Full reproduction of the entire surface was obtained by using periodic boundary conditions on the planes that are vertical to *x* and *y*, and perfectly matched layer boundary conditions on the sides that are vertical to *z*. In our analysis, we use a nonuniform spatial grid with sizes varying from 1 nm in the *z* direction to 10 nm in the *x* and *y* directions. The DFLC mixture was described by the permittivities and the conductivities. For a low frequency equal to f_L_ = 1 kHz, they were set as ε_II_ = 10.4, ε_⊥_ = 7.4, σ_II_ = 0.71·10^−6^ S/m, and σ_⊥_ = 0.52·10^−6^ S/m, and for a high frequency of f_H_ = 40 kHz they were equal to ε_II_ = 4.1, ε_⊥_ = 7.1, σ_II_ = 0.38·10^−6^ S/m, and σ_⊥_ = 0.47·10^−6^ S/m. Metallic elements were modeled using the Drude model of electrical conduction. Dielectric substrates are considered to be lossless with a constant refractive index of 2.12. Following these assumptions, we can simulate the tunable response of the 3D meta-device depicted in Fig. 1(a–e), assuming that an incident electromagnetic wave illuminating the meta-cell propagates along the *z* direction, as shown in Fig. 1(d).

### Metamaterial fabrication

To fabricate a metamaterial nanostructure, we used an electron beam lithography system based on a scanning electron microscope, specifically Jeol 6400 with LaB_6 filament and maximum acceleration voltage of 40 keV. The microscope was equipped with an ELPHY Plus 16-bit pattern processor and a high-precision, laser-interferometric positioning stage (resolution 5 nm), which provided the “reference” coordinate system. The standard methods of e-beam lithography, like area scanning or single-dot exposure, cannot be applied here because element-by-element patterning takes additional time (the so-called settling time). Therefore, fabrication of 10^8^ elements with a standard e-beam system requires more than 24 h, which is not acceptable. We solved this technological problem by designing a special one-element structure, which was patterned in the single-line exposure mode. An additional problem, which was related to the charging of the resist material and pattern displacement, was solved by the application of a conductive polymer film.

The special one-element structure was designed in the form of a line, meandering from left-to-right and right-to-left, in which the individual parallel segments were 160 nm distant. This single-line element was irradiated with the exposure step size of 160 nm. In this way, a regular array of points was obtained. The final size of each dot was determined by the exposure dose, which was obtained experimentally. This approach eliminated the setting time delay and ensured that the total exposure time was reduced to approximately 4 h. The line pattern covered a writing field measuring 163.84 μm × 163.84 μm and was exposed side by side, within a 2-mm × 2-mm range, using stage movement with the position controlled by the laser interferometer.

For the e-beam-sensitive resist, we used the high-resolution and high-contrast material CSAR-62 (Allresist GmbH) spun to a thickness of 100 nm. Resist layers were subsequently covered with ≈ 40nm of conductive coating Electra-92 (Allresist GmbH), which effectively dissipated electric charges from our insulating substrates. Exposure procedures were conducted with the electron energy set to 30 keV and a beam current of 0.1 nA. After the development, patterned samples were covered with 4 nm of Cr and 16 nm of Au (thermal evaporator). For lift-off processing, we used the dedicated CSAR-62 remover (AR600-71), which gave much better results than the application of a standard solvent (acetone).

### Atomic force microscopy

The metasurface topography was characterized using the fast-scanning atomic force microscope Cypher (Oxford Asylum Research), equipped with standard micro cantilever OMCL-AC160TS-R3 (Olympus, Oxford Instruments). The OMCL-AC series has a tetrahedral tip on the exact end of the cantilever. The nominal probe spring constant was equal to 26.1 N/m and the tip radius was 7 nm. Maps visualizing the metamaterial nanostructure were obtained by adopting a contact mode, and then they were processed graphically by employing the IGOR Pro ver. 6.32 A software prepared by the AFM manufacturer. Furthermore, to obtain a full range of colors in addition to highlighting the architecture of nano-elements, the 3D image level was slightly lowered, which is why we see negative values on the scale showing the height.

### DFLC synthesis

Multicomponent liquid crystalline mixture 1909C, exhibiting dual-frequency behavior with a cross-over frequency at 12.6 kHz and ɛ_L_ =  + 3 and ɛ_H_ = −3 was designed and synthesized. The perpendicular component of electric permittivity varied between 7.5 and 6.9 for low and high frequencies, respectively. The mixture is composed of two types of components from a few families of nematic materials. Dielectrically positive isothiocyanato tri-ring tolanes used for this mixture possess birefringence equal to 0.42 and relaxation frequency measured at 20 °C on the level of 20–30 kHz. Dielectrically negative derivatives of multifluorinated dialkyl or alkyl-alkoxy tolanes and phenyltolanes have birefringence about 0.27 and electric permittivity anisotropy below −5^47–49^. The ratio between these two groups of components was chosen to ensure symmetric dielectric anisotropies and low cross-over frequency at the same time. To ensure uniform alignment and switching of DFLC, a small amount (0.2%) of CB15 type chiral dopant was added.

### Spectral characterization

The spectral characterization of the metadevice was made based on a measuring system that was built on the basis of the supercontinuum laser SC450-4-AOTF (Fianium, NKT Photonics) and optical spectrum analyzer AQ6373B (Yokogawa) with wavelength accuracy of ± 0.05 nm. Light from a supercontinuum source was coupled to a standard telecommunication single-mode fiber SMF28 (Corning) to illuminate a metamaterial cell. Also, the same kind of a fiber was used for providing a signal to the optical spectrum analyzer. The SMF28 series has a core diameter equal to 8 µm and a numerical aperture NA = 0.14. To ensure precise manipulation of the fiber position in relation to the DFLC-loaded metamaterial cell, the 3DMAX system with piezomechanical controls from Thorlabs was used. The measurement system also included the waveform generator DG1022 (Rigol) and the linear voltage amplifier F10AD (FLC Electronics).

### Tests of switching times

Switching time measurements of the DFLC-loaded metamaterial device were performed by employing a system consisting of the BIOLAR PI polarizing microscope 33120 A (Hewlett Packard), function/arbitrary waveform generator, linear voltage amplifier F10AD (FLC Electronics), photodetector PIN 20 (FLC Electronics), and Tektronix TDS 2014 oscilloscope.
